# Interrater reliability in the assessment of oral mucositis among patients receiving high-dose chemotherapy: a multicenter comparison between specialized dentists and registered nurses

**DOI:** 10.1186/s12885-025-14670-3

**Published:** 2025-12-17

**Authors:** Aram Ibrahim, Dilav Mahmoud, Hanna Mavandadipur, Java Walladbegi

**Affiliations:** Department of Oral and Maxillofacial Surgery, Institute of Odontology, The Sahlgrenska Academy, University of Gothenburg, PO Box 450, Göteborg, 405 30 Sweden

**Keywords:** Cryotherapy, Lymphoma, Multiple myeloma, Oral mucositis, WHO toxicity scale

## Abstract

**Background:**

Accurate diagnosis of oral mucositis (OM) is essential for effective management and plays a critical role in ensuring reliable outcomes in clinical trials evaluating OM-prevention and treatment strategies. Currently, no evidence-based guidelines specify which clinical profession should be primarily responsible for OM assessments in patients undergoing high-dose chemotherapy. In most Scandinavian countries, nurses perform daily OM assessments, while dentists specialized in orofacial medicine (considered the gold standard) conduct supplementary evaluations several times per week. However, the concordance between nurses’ assessments and those conducted by dentists specialized in orofacial medicine remains unclear. Therefore, this study aimed to evaluate the interrater reliability in of OM assessments between these two professions.

**Methods:**

This study utilized data from a randomized, blinded, multicenter, parallel-group, phase 3 trial conducted at five university hospitals in Sweden and Norway. A total of 127 patients aged 18 years or older, diagnosed with multiple myeloma or lymphoma, and scheduled to receive high-dose conditioning chemotherapy in preparation for hematopoietic stem cell transplantation, were included. The primary objective was to evaluate the interrater reliability of OM assessments across all severity grades using the World Health Organization (WHO) oral toxicity scale (grades 0–4). The secondary objective was to evaluate the interrater reliability of OM assessments across all severity grades at each individual study site.

**Results:**

Overall interrater reliability between nurses and dentists specialized in orofacial was found to be fair for OM of any grade (κ = 0.211, *p* < 0.001). However, agreement levels at individual study sites ranged from no agreement to fair agreement.

**Conclusion:**

The interrater reliability in the assessment of oral mucositis between dentists specialized in orofacial medicine and nurses ranged from no-, to fair agreement. This highlights the need for targeted interventions to improve nurses' assessment skills or to integrate a specialized dentist as a permanent member of the multidisciplinary team in oncology and hematology settings.

**Trial registration** This study is based on data from a previously registered clinical trial (ClinicalTrials.gov; Identifier: NCT03203733, Registered on November 20, 2020).

## Introduction

Oral mucositis (OM) affects up to 80% of patients undergoing high-dose chemotherapy (HDCT) in preparation for hematopoietic stem cell transplantation (HSCT) [1], usually developing within the first week following HDCT. Clinically, OM presents with a range of manifestations, from erythema to patchy or confluent ulcerations, often covered by a superficial pseudomembrane [2]. These lesions are frequently accompanied by a variety of debilitating symptoms, including dysphagia, difficulty in eating and drinking, and severe oral pain, which may necessitate increased use of analgesics, including intravenous opioids in severe cases [3, 4].

HDCT combined with HSCT is an established and effective treatment for several hematological malignancies, including multiple myeloma and lymphoma [5, 6]. However, the non-selective nature of chemotherapeutic agents, which target both malignant and rapidly dividing healthy cells, poses a significant challenge by exposing patients to various adverse effects, among which OM is one of the most prominent [7, 8].

Diagnosis of OM typically relies on patient-reported symptoms alongside clinical oral examination. Several validated tools exist to record the extent and severity of OM, with the World Health Organization (WHO) oral toxicity scale being among the most widely used. The WHO scale combines both objective clinical findings and subjective symptomatology, making it accessible for use by a variety of healthcare professionals in clinical settings [9, 10].

Despite its clinical relevance, no evidence-based guidelines currently specify which clinical healthcare profession should be primarily responsible for OM assessments in patients receiving HDCT. As a result, assessments practices vary across hospitals and countries. In most Scandinavian countries, nurses perform daily OM assessments as part of routine care, while dentists specialized in orofacial medicine conduct additional assessments several times per week. However, it remains unclear whether nurses’ assessments are comparable in reliability and accuracy to those performed by dentists specialized in orofacial medicine. Therefore, the aim of this study was to investigate the interrater reliability in the assessment of OM between these two professions.

## Patients and methods

### Study design

This study utilized data collected from a randomized, blinded, multicenter, parallel-group, phase 3 trial conducted across five university hospitals in Sweden and Norway: Uppsala University Hospital; Karolinska University Hospital; Linköping University Hospital; Örebro University Hospital; and Oslo University Hospital [11, 12].

### Patients

Eligible patients were adults aged 18 years or older with a confirmed diagnosis of multiple myeloma or lymphoma, scheduled to receive high-dose conditioning chemotherapy prior to HSCT, were enrolled in this study. Eligibility criteria included suitability for high-dose melphalan (multiple myeloma patients) or a high-dose BEAC or BEAM regimens (lymphoma patients), as determined by the investigator. Patients were also required to be proficient in the primary language of the respective center (Swedish or Norwegian) and capable of understanding both oral and written information related to the study. Patients who were unable to comprehend the study information were excluded. Additional exclusion criteria included concurrent participation in other clinical trials that, in the investigator’s judgment, could interfere with study outcomes, or if post-transplant follow-up was expected to occur at hospitals outside the regions of the participating study centers.

### Conditioning therapy

Conditioning high-dose chemotherapy, infection prophylaxis during cytopenia, and supportive care were administered according to institutional protocols. All patients with multiple myeloma received a single intravenous dose of Melphalan (140 mg/m^2^ or 200 mg/m^2^) 24 h prior to HSCT. Patients with lymphoma underwent conditioning with either BEAC or BEAM high-dose chemotherapy regimen [13, 14]. Most patients received autologous peripheral blood-derived multipotent hematopoietic stem cells, collected following chemotherapy and routine mobilization with granulocyte-colony stimulating factor (G-CSF; Filgrastim) targeting a minimum yield of ≥ 2 × 10⁶ CD34^+^ cells/kg. Following HSCT, Filgrastim or Lipegfilgrastim was administered in accordance with site-specific clinical protocols [12].

### Oral mucositis assessment

As part of a Clinical Assistance Program, dental staff specialized in orofacial medicine (considered the gold standard for OM assessment) were calibrated to evaluate and document the severity of OM. To assess interrater reproducibility among the dental staff, an intraclass correlation coefficient (ICC) analysis was performed on a sample dataset using the WHO oral toxicity scale. The results demonstrated moderate reliability (ICC = 0.672; 95% CI: 0.007–0.927; *p* = 0.026). Within the same program, nursing staff also received structured training in the assessment of OM severity.

OM was systematically evaluated at eight intraoral locations: the buccal-, and palatal mucosae, lips, floor of the mouth, tongue, and gingiva. Assessments were conducted using the WHO scale, which ranges from grade 0 to 4 (0 = Healthy mucosa; 1 = Oral soreness and erythema; 2 = Erythema and ulcers, but the patient can swallow solid food; 3 = Ulcers with extensive erythema, patient cannot swallow food; 4 = Mucositis severe enough to prevent alimentation).

Each patient was assessed three times per week (Monday, Wednesday, Friday) by both professions, though not at the same time on any given day. Assessments began at admission and continuing until discharge, i.e., when OM had resolved or until day + 28 post-HSCT, a timeframe generally considered sufficient to capture the critical phases of mucosal damage and recovery. For analysis, the highest OM score recorded by a single orofacial medicine specialist during each patient’s care was compared with the score recorded by one nurse. The same orofacial medicine specialist conducted all dentist assessments, whereas the nurse performing the assessment could vary across time points.

### Data collection

Clinical data were collected using paper-based checklists, each identified by a pre-printed trial number and a unique combination of patient study numbers assigned at registration. An authorized medical staff member completed the checklist on site, with each form dated and signed by the responsible physician upon completion. The PheedIt-system was used for data capture and served as the clinical database for the study. Uppsala Clinical Research was responsible for establishing the clinical database, providing technical support, programming logical computerized checks, preparing the Data Management Plan, and overseeing the management process for the PheedIt-system. Data from the checklists were entered, cleaned, and validated by a designated individual at each study site, who had signed a confidentiality agreement. Entered data were subjected to logical computerized validation checks, and any discrepancies identified were reviewed by the designated data manager. If corrections were required, they were documented on a Data Clarification Form (DCF), which was signed and dated by the responsible physician. All updates to the clinical database were audited, and any corrections made to the data were recorded accordingly. The original paper checklists were retained on site as source documents, while copies were collected by the designated data manager. For the purposes of this study, data specific to OM assessments using the WHO scale were extracted from these checklists. Data retrieval was conducted by two independent investigators (AI, JW). Prior to data extraction, both investigators underwent calibration to standardize their search strategy, minimize missing data, and ensure alignment with the overall study design.

### Objectives

*Primary objective:*

To evaluate the interrater reliability of OM assessments across all severity grades using the WHO oral toxicity scale (grades 0–4).

*Secondary objective:*

To evaluate the interrater reliability of OM assessments using the WHO oral toxicity scale (grades 0–4).

across all severity grades at each individual study site.

### Statistical analysis

To assess interrater reliability, Cohen’s Kappa (κ) statistics was used. The interpretation of κ values was as follows: ≤0 indicates no agreement, 0.01–0.20, slight agreement, 0.21–0.40 indicates fair agreement, 0.41–0.60 denotes moderate agreement, 0.61–0.80, substantial agreement, and 0.81–1.00, almost perfect agreement [15]. Wilcoxon signed-rank test was used to determine statistically significant differences between OM severity ratings provided by the orofacial medicine specialist and the nurse for each specific assessment. A *p*-value of ≤ 0.05 was considered statistically significant. All statistical analyses were performed using the IBM^®^ SPSS^®^ Statistics software package, version 26 (IBM Corp., Armonk, NY, USA).

## Results

A total of 127 out of the 182 patients initially enrolled in the randomized, blinded, multicenter, parallel-group, phase 3 trial had complete data available for inclusion in this study. For the remaining 55 patients, clinical records related to OM assessments using the WHO toxicity scale were missing. Reasons for missing data included disease-related mortality (*n* = 2), withdrawal of consent (*n* = 9) in accordance with ethical guidelines allowing patients to withdraw at any given time without providing a reason, and lack of post-treatment follow-up (*n* = 44). The majority of missing records originated from Karolinska University Hospital (*n* = 52) and Örebro University Hospital (*n* = 3). Uppsala University Hospital included patients with either multiple myeloma or lymphoma, while the other four study sites enrolled only patients with multiple myeloma. Most patients underwent their first HSCT during the study (*n* = 102/127), whereas a smaller proportion had previously received HSCT once (*n* = 23/127) or twice (*n* = 2/127). A summary of study sites and patient characteristics is presented in Table 1.

**Table 1:** Summary of study sites (University Hospitals) and patient characteristics

**Study sites [University Hospitals]**	**Total No. of patients [** ***n*** ** = 127]**	
Uppsala	70	
Karolinska	14	
Linköping	25	
Örebro	11	
Oslo	7	
**Gender [F/M]**	48	79
**Age, years [**$\bf\:\bar{\textbf{x}}$¯**±SD]**	58 ± 9	63 ± 8
**Diagnoses**		
*Multiple myeloma [Total]*	[101]	
Conditioning chemotherapy		
Melphalan 140 mg/m^2^	2	
Melphalan 200 mg/m^2^	99	
*Lymphoma [Total]*	[26]	
*Non-Hodgkin’s	20	
Mantle Cell	8	
Diffuse large B–Cell	7	
Follicular	3	
High grade B–Cell	2	
*Hodgkin’s	2	
T–Cell	2	
Other atypical subtypes	2	
Conditioning chemotherapy		
BEAC	23	
BEAM	3	
**HSCT**		
I	102	
II	23	
III	2	

High-dose chemotherapy regimens for lymphoma: BEAC (carmustine; cytarabine; etoposide; cyclophosphamide) and BEAM (carmustine; cytarabine; etoposide; melphalan). *B-cell lymphomas. HSCT = Hematopoietic stem cell transplantation. I = first transplantation; II = one transplantation prior to the study; III = two transplantations prior to the study

Table 2 provides an overview of the total number of OM assessments (*n* = 127) performed by both dental-, and nursing staff. The highest level of agreement between the two professions was observed for OM grade 0 (41/65; 63%). In contrast, agreement was considerably lower for more severe grades, grade 3 (1/9; 11%) and grade 4 (0/2; 0%).

**Table 2:** Oral mucositis assessments and agreements between dentist specialized in orofacial medicine and nurses

		Dental specialists					
		Grade 0	Grade 1	Grade 2	Grade 3	Grade 4	Total
Nurses	Grade 0	41	12	1	2	0	56
	Grade 1	15	7	7	0	1	30
	Grade 2	6	7	10	3	0	26
	Grade 3	3	3	2	1	1	10
	Grade 4	0	0	2	3	0	5
	Total	65	29	22	9	2	127
κ = 0.211, i.e., fair agreement, *p* < 0.001							

Cross-tabulations presenting the total number of oral mucositis cases assessed across all severity grades (WHO–scale; grades 0–4) by dentist specialized in orofacial medicine and registered nurses, as well as the number of cases where the assessments were in agreement. κ = Cohen’s Kappa. A *p*-value ≤ 0.05 was considered statistically significant

Regarding the primary objective, i.e., OM assessment across all severity grades using the WHO toxicity scale, the interrater reliability between the two professions was considered as fair (κ = 0.211, *p* < 0.001).

For the secondary objective, i.e., OM assessment across all severity grades at each individual study site, the interrater reliability between the two professions was as follows: Uppsala University Hospital (κ = 0.276, fair agreement, *p* < 0.001); Karolinska University Hospital (κ = 0.133, none to slight agreement, *p* = 0.443); Linköping University Hospital (κ=-0.031, no agreement, *p* = 0.804); Örebro University Hospital (κ = 0.298, fair agreement, *p* = 0.165); and Oslo University Hospital (κ=-0.061, no agreement, *p* = 0.803). The distribution of OM assessment scores and corresponding Cohen’s Kappa (κ) values for each study site are presented in Table 3.

**Table 3:** Oral mucositis assessments and agreements between dentist specialized in orofacial medicine and nurses at each individual study site

			Dental specialists					
Uni. Hospitals			Grade 0	Grade 1	Grade 2	Grade 3	Grade 4	Total
Uppsala [κ = 0.276] *p* < 0.001	**Nurses**	Grade 0	19	7	0	1	0	27
		1	3	4	5	0	1	13
		2	3	5	9	2	0	19
		3	1	3	1	1	1	7
		4	0	0	2	2	0	4
		Total	26	19	17	6	2	70
Karolinska [κ = 0.133] *p* = 0.443	**Nurses**	Grade 0	5	1	1	1	–	8
		1	2	1	0	0	–	3
		2	1	0	1	0	–	2
		3	1	0	0	0	–	1
		4	–	–	–	–	–	–
		Total	9	2	2	1	–	14
Linköping [κ=-0.031] *p* = 0.804	**Nurses**	Grade 0	8	4	0	0	–	12
		1	5	1	1	0	–	7
		2	2	0	0	1	–	3
		3	1	0	1	0	–	2
		4	0	0	0	1	–	–
		Total	16	5	2	2	–	25
Örebro [κ = 0.298] *p* = 0.165	**Nurses**	Grade 0	7	0	–	–	–	7
		1	3	1	–	–	–	4
		2	–	–	–	–	–	–
		3	–	–	–	–	–	–
		4	–	–	–	–	–	–
		Total	10	1	–	–	–	11
Oslo [κ=-0.061] *p* = 0.803	**Nurses**	Grade 0	2	0	0	–	–	2
		1	2	0	1	–	–	3
		2	0	2	0	–	–	2
		3	–	–	–	–	–	–
		4	–	–	–	–	–	–
		Total	4	2	1	–	–	7

Cross-tabulations presenting the total number of oral mucositis cases assessed across all severity grades (WHO–scale; grades 0–4) by dentist specialized in orofacial medicine and registered nurses at each individual study site, as well as the number of cases where the assessments were in agreement. The Cohen’s Kappa (κ) values are presented in brackets for Uppsala-, Karolinska-, Linköping-, Örebro-, and Oslo university hospitals. A *p*-value ≤ 0.05 was considered statistically significant

## Discussion

In most Scandinavian countries, the nursing profession continues to oversee the daily assessment of chemotherapy-induced OM. However, it has not been previously evaluated whether these assessments are comparable to those conducted by dentists specialized in orofacial medicine. This gap in knowledge provided the rationale for conducting the present study.

The interrater reliability in OM assessments across all severity grades demonstrated a fair level of agreement between the two professions. These findings contrast with a previous study that reported high interrater reliability in OM assessments across different healthcare professions [16]. Notably, in that study, assessments were consistently performed by the same health care professionals, all of whom had extensive experience in evaluating the oral mucosa of patients at high risk for OM. This was not the case in our study, particularly among the participating nurses. Moreover, there is a limited number of studies evaluating the level of agreement in OM assessments conducted by different healthcare professionals, and a lack of standardized reference criteria further complicates the validation of our findings.

Given these results, one could argue that specialists in orofacial medicine should assume primary responsibility for OM assessments in clinical settings. However, as the specialists typically operate on a consultation basis, with an estimated cost of USD 230 for the initial consultation and USD 45 for follow-up visits (personal communication), the financial sustainability of this approach remains uncertain for some institutions.

In such cases, implementing measures aimed at enhancing the diagnostic capabilities of nursing staff may be more pragmatic solution for minimizing diagnostic errors. Furthermore, greater resources should be directed toward bridging the knowledge gap between the two professions. Strategies such as continuous education, professional development through current literature and research, participation in conferences and workshops, mentorship programs, structured training curricula and decision-support tools could significantly enhance nurses’ knowledge and competency in the management of OM [17–19]. Nevertheless, the authors advocate for the integration of a trained dentist specialized in orofacial medicine as a permanent member of the multidisciplinary team in oncology and hematology centers. This approach, rather than relying on episodic clinical support, would not only improve diagnostic accuracy and enable early intervention for OM but also enhance overall oral health outcomes for immunocompromised patients, thereby reducing the risk of patient suffering. Additionally, it may prove more cost-effective in the long term by reducing the incidence of complications and the associated need for more intensive treatments.

When interrater reliability in OM assessments across all severity grades was evaluated at each individual study site, the level of agreement between the two professions was generally poor. As most of the observations did not reach statistical significance, caution is warranted when interpreting these results. Nevertheless, these findings are noteworthy, as diagnostic discrepancies may lead to downstream consequences, potentially resulting in significant harm to patients and adversely affecting their quality of life [20].

A potential explanation for the poor interrater agreement may be the limited emphasis on oral health-related conditions in nursing education programs, where such topics are addressed to a significantly lesser extent compared to medical and dental programs. This educational gap may contribute to reduced confidence among nurses in recognizing and assessing oral pathologies. Furthermore, only approximately 50% of nurses have received any formal training in OM management during their careers, and just 10% have received more than five hours of training in this area [21]. Previous studies have highlighted that oral health assessments and care practices within the nursing profession are often suboptimal [22], particularly when using standardized protocols related to OM [23, 24]. In fact, research conducted among oncology nurses have revealed insufficient knowledge regarding oral pathologies, including the assessment, grading, and management of OM [23, 25]. In contrast, specialists in orofacial medicine complete a three-year hospital-based residency program, during which they develop proficiency in managing complex conditions of the orofacial region and gain a comprehensive understanding of the interactions between systemic diseases, medical treatments, and oral conditions. This includes specialized expertise in managing chemotherapy-induced OM, particularly in patients undergoing HSCT [26].

Although this was a multicenter study with a large sample size across several sites in Sweden and Norway, certain limitations should be acknowledged. First, the orofacial medicine specialists responsible for OM assessments and the overall conduct of the study remained consistent throughout the trial, whereas the nursing staff varied over time. Such variability is difficult to avoid in long-term studies, particularly in clinical settings with high staff turnover. In future research, it would be advantageous to limit the number of nurse evaluators and ensure that the same individuals are predetermined for participation, as was done with the specialist dentists.

Second, the study included a relatively small number of patients at high risk of developing severe OM, such as those with lymphoma [12]. Moreover, most of the lymphoma patients in this study were conditioned with BEAC, a chemotherapeutic regimen associated with lower severity of OM compared to BEAM [27]. Additionally, all patients received cryoprevention, a modality known to reduce the incidence and severity of OM [28]. Given that our results showed higher interrater agreement in cases of absent or low-grade OM, and greater variability as OM severity increased, these factors may have influenced the study outcomes and limited the detection of statistically significant differences.

Standardizing OM assessments in clinical settings presents both challenges and significant opportunities for improving patient care. A key practical implication is ensuring that assessment tools are sensitive, reliable, and adaptable across diverse patient populations, particularly in oncology and hematology where OM is prevalent. Consistent use of validated OM assessment instruments in both clinical trials and routine care could improve early detection, severity grading, and management strategies. Looking ahead, large-scale multicenter validation studies, including study arms without cryoprevention, where ethically feasible, are essential to confirm the reliability and generalizability of these tools in varied clinical contexts. Ultimately, standardized OM assessments can improve the precision of interventions, leading to better patient outcomes and more effective resource allocation [29, 30].

## Conclusion

The interrater reliability in the assessment of oral mucositis between dentists specialized in orofacial medicine and nurses ranged from no-, to fair agreement. This highlights the need for targeted interventions to improve nurses' assessment skills or to integrate a specialized dentist as a permanent member of the multidisciplinary team in oncology and hematology settings.

## Data Availability

The data that support the findings of this study are available from the BrainCool AB but restrictions apply to the availability of these data, which were used under license for the current study, and so are not publicly available. Data are however available from the authors upon reasonable request and with permission of BrainCool AB.

## Abbreviations

HDCT: High-dose chemotherapy
HSCT: Hematopoietic stem cell transplantation
OM: Oral mucositis
WHO: World Health Organization
